# Electrochemical Determination of Capsaicinoids Content in Soy Sauce and Pot-Roast Meat Products Based on Glassy Carbon Electrode Modified with Β-Cyclodextrin/Carboxylated Multi-Wall Carbon Nanotubes

**DOI:** 10.3390/foods10081743

**Published:** 2021-07-29

**Authors:** Qianhui Gu, Chaoqun Lu, Kangwen Chen, Xingguang Chen, Pengfei Ma, Zhouping Wang, Baocai Xu

**Affiliations:** 1 State Key Laboratory of Food Science and Technology, School of Food Science and Technology, Jiangnan University, 1800 Lihu Ave, Wuxi 214122, China; guqianhui25@126.com (Q.G.); babystarchen@163.com (X.C.); mapf2011@163.com (P.M.); 2 Three Squirrels Inc., 8 Jiusheng Road, Wuhu 241000, China; luchaoqun1104@163.com (C.L.); r_smran@3songshu.com (K.C.); 3 Engineering Research Center of Bio-Process of Ministry of Education, School of Food and Biological Engineering, Hefei University of Technology, 420 Feicui Road, Hefei 230601, China

**Keywords:** capsaicinoids content, soy sauce and pot-roast meat products, electrochemical sensor, β-cyclodextrin, carboxylated multi-wall carbon nanotubes

## Abstract

The rapid quantification of capsaicinoids content is very important for the standardization of pungent taste degree and flavor control of soy sauce and pot-roast meat products. To rapidly quantify the capsaicinoids content in soy sauce and pot-roast meat products, an electrochemical sensor based on β-cyclodextrin/carboxylated multi-wall carbon nanotubes was constructed and the adsorptive stripping voltammetry method was used to enrich samples in this study. The results showed that the excellent performance of the established electrochemical sensor was mostly because β-cyclodextrin caused the relative dispersion of carboxylated multi-wall carbon nanotubes on the glassy carbon electrode surface. Capsaicin and dihydrocapsaicin had similar electrochemical behavior, so the proposed method could determine the total content of capsaicinoids. The linearity of capsaicinoids content was from 0.5 to 100 μmol/L and the detection limit was 0.27 μmol/L. The recovery rates of different capsaicinoids content were between 83.20% and 136.26%, indicating the proposed sensor could realize trace detection of capsaicinoids content in sauce and pot-roast meat products. This work provides a research basis for pungent taste degree standardization and flavor control in the food industry.

## 1. Introduction

Soy sauce and pot-roast meat products are traditional Chinese cooked meat products with a unique flavor and a delicious taste. Among many elements of taste, the pungent taste is one of the consumer’s favorite flavors [1]. However, there is no uniform standard to evaluate the pungent taste degree of sauce and pot-roast meat products, so it is difficult to provide guidance for product production and customer selection. Capsaicinoids are a group of lipophilic alkaloids that are responsible for the pungent taste in most foods, whose content is often used to calculate the pungent taste degree of food. Therefore, the determination of capsaicinoids content is the key to the evaluation of the pungent taste degree.

At present, many analytical methods for the determination of capsaicinoids content have been developed, such as high performance liquid chromatography [2], gas chromatography [3], gas chromatography with mass spectrometry [4], and liquid chromatography with mass spectrometry [5]. Although these methods can accurately and sensitively detect the capsaicinoids content, the requirement of expensive instruments and experienced operators limits the wide application of these methods [6]. Therefore, the development of a simple, rapid, and low-cost method for the detection of capsaicinoids content is the key to the analysis of pungent taste degree.

In recent years, with the development of analytical techniques, many new analytical methods of capsaicinoids have been developed, including ELISA [7], infrared spectroscopy [8], electrochemical methods, etc. Compared with ELISA and infrared spectroscopy, electrochemical detection is recognized as a more economical and easier to miniaturize method [9,10,11,12,13]. To meet the determination of capsaicinoids content, electrochemical sensors based on carbon nanotubes (CNTs) [14,15], graphene [16,17], and nanoparticles [18] have been developed. Among them, electrochemical sensors modified with CNTs are promising candidates [19,20,21,22]. Kachoosangi et al. [23] prepared an electrochemical sensor for detecting capsaicin content with a detection limit of 0.31 mol/L using a pyrolytic graphite electrode modified with a multi-wall carbon nanotube (MWCNT). Ziyatdinova et al. [24] used a glassy carbon electrode (GCE) modified with carboxylated single-walled carbon nanotubes (SWCNTs-COOH) and CeO_2_-surfactants dispersions to detect capsaicin content and obtained good agreement with the UV-spectroscopic data. Although CNTs have excellent performance in detecting capsaicinoids content, whose poor water dispersibility can bring great limitation to the use [25]. β-cyclodextrin (β-CD) are environmentally friendly and water-soluble and can improve the solubility and stability of CNTs. Therefore, β-CD has been widely used in the modification of CNTs. Liu et al. [26] prepared carbon paper modified with β-CD/CNTs and realized the rapid and sensitive detection of methyl parathion content. Ali et al. [27] used a screen-printed carbon electrode modified with β-CD/CNTS to detect bisphenol A content in water. In addition, it has been reported that β-CD has certain sensitization effect on capsaicinoids content detection [28]. Therefore, we speculated that the β-CD/CNTS composite material is suitable for the electrochemical detection of capsaicinoids content. However, there are few reports on the detection of capsaicinoids content in soy sauce and pot-roast meat products using electrodes modified with β-CD/CNTs.

Differential pulse voltammetry (DPV) is a powerful electroanalytical technique, which has attracted much attention due to its advantages of simple operation and low cost. Adsorptive stripping voltammetry (AdSV) is a rapid and sensitive electrochemical analytical method, which is mainly used to improve the sensitivity of the determination of metal ions and organic molecules in environmental water samples. These two techniques are often used together in electrochemical measurements to improve the detection effect [29]. Lyu et al. [6] reported the electrochemical detection of capsaicinoids content using a differential pulse adsorptive stripping voltammetry (DP-AdSV) method using unmodified screen-printed carbon electrodes, which provided to be a promising tool in the food industry.

In this study, an electrochemical sensor based on β-CD and carboxylated multi-wall carbon nanotubes (MWCNT-COOH) combined with the DP-AdSV method was constructed to achieve the simple, fast, and economic detection of capsaicinoids content in soy sauce and pot-roast meat products. Our observations demonstrated that the sensor modified with β-CD and MWCNT-COOH could bring new promotion for detecting capsaicinoids content in soy sauce and pot-roast meat products. This study is of great significance to the standardization of pungent taste degree and flavor control in the food industry.

## 2. Materials and Methods

### 2.1. Materials and Instrumentation

All reagents used were of analytical grade. MWCNT-COOH was purchased from Nanjing XFNANO Materials Tech Co., Ltd. (Nanjing, Jiangsu, China). Cholesterol, glucose, sucrose, β-CD, hydrochloric acid (HCl), anhydrous ethanol, methanol, anhydrous sodium acetate, potassium chloride, potassium ferrocyanide, and potassium ferricyanide were purchased from Sinopharm Chemical Regent Co., Ltd. (Shanghai, China). Capsaicin and dihydrocapsaicin were purchased from Chengdu Must Bio-technology Co., Ltd. (Chengdu, Sichuan, China). Slurries powder (0.05 μm) and all electrodes was purchased from Shanghai Xianren Instrument Co., Ltd. (Shanghai, China). The ultrapure water was prepared by distillation and then purified with a water purification system (Sichuan Youpu Ultrapure Science and Technology Co., Ltd. (Chengdu, Sichuan, China)). Soy sauce and pot-roast duck neck, soy sauce and pot-roast chicken claw, and soy sauce and pot-roast beef were obtained by Three Squirrels Inc (Wuhu, Anhui, China). Preparation of 10 mmol/L [Fe(CN)_6_]^3−/4−^ solution: 18.64 g of potassium chloride, 0.41 g of potassium ferrocyanide, and 0.53 g of potassium ferrocyanide were weighed and dissolved in 250 mL of deionized water.

Electrochemical experiments were performed on an LK2010 electrochemical workstation, which was purchased from Tianjin Lanlike Chemical Electronics High-tech Co., Ltd. (Tianjin, China). All the electrochemical experiments (cyclic voltammetry, DPV, and DP-AdSV method) were performed in a three-electrode system (A GCE or modified GCE was used as the working electrode, a saturated calomel electrode was used as the reference electrode, and a platinum electrode was used as counter electrode).

### 2.2. Preparation and Characterizations of Modified Electrodes

**Scheme 1:**
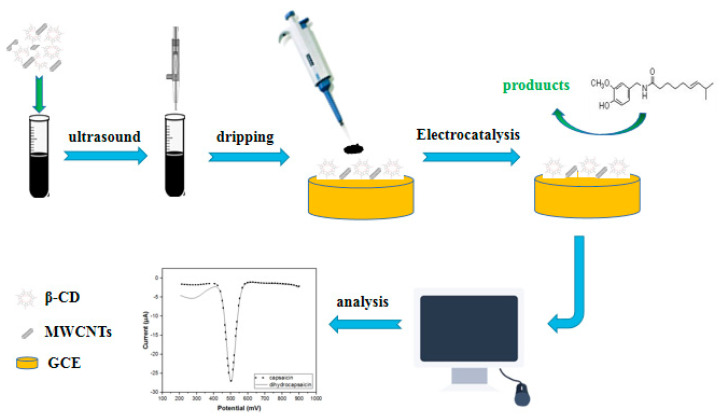
The preparation process of β-CD/MWCNT-COOH/GCE.

The preparation process of β-CD/MWCNT-COOH/GCE (β-GCNT) is shown in Scheme 1. Before starting each experiment, the GCE was polished using 0.05 μm of alumina slurries powder and ultrasonically washed for 3 min in ethanol and ultrapure water, respectively. A total of 200 mg of β-CD and 20 mg of MWCNT-COOH were dispersed with the aid of ultrasonic agitation in 10 mL ultrapure water to give a black dispersion solution. The β-GCNT was prepared by dropping this dispersion (1.7 μL) on GCE surface and then dried to remove the solvent [30,31]. β-CD/GCE (β-GCE) and MWCNT-COOH/GCE (GCNT) were also prepared by a similar method. 

The micromorphology of GCE, GCNT, β-GCE, and β-GCNT were obtained by scanning electron microscopy (SEM, FEI inspect F50). The static contact angles of GCE, GCNT, β-GCE, and β-GCNT were obtained by static contact angle measuring instrument (SDC-200). The electrochemical characterization of GCE, GCNT, β-GCE, and β-GCNT was obtained by scanning 10 mmol/L of [Fe(CN)_6_]^3−/4−^ solution using cyclic voltammetry (CV) method [10,31]. The initial parameter of CV for scanning [Fe(CN)_6_]^3−/4−^ solution: the potential range was −200~600 mV and the scanning rate was 100 mV/s. To obtain the charge transfer number of different electrodes, 10 mmol/L of [Fe(CN)_6_]^3−/4−^ solution was scanned by the cyclic voltammetry (CV) method with GCE, GCNT, β-GCE, and β-GCNT. In order to further clarify the electrochemical control process of [Fe(CN)_6_]^3−/4−^ solution on β-GCNT’s surface, 10 mmol/L of [Fe(CN)_6_]^3−/4−^ solution was scanned under different CV scanning rates (20, 50, 100, 200, 300, 400, 500, and 600 mV/s). All experiments were carried out three times.

### 2.3. Study on Electrochemical Behavior of Capsaicinoids

The electrochemical behavior of capsaicin on β-GCNT sensors was studied by the CV and DPV method. The initial parameters of CV: the potential range was 0 ~ 1000 mV and the scanning rate was 100 mV/s. The initial parameters of DPV: the potential range was 200~900 mV, impulse amplitude was 50 mV, pulse width was 200 ms, pulse period was 400 ms, and equilibrium time was 2 s. 0.1 mol/L of HCl solution was used as the initial supporting electrolyte and β-GCNT was used as the working electrode. 

In order to obtain the number of electrons involved in the electrochemical reaction, 50 μmol/L capsaicin solution was scanned under different CV scanning rates (20, 100, 200, and 300 mV/s) [10]. To obtain the number of protons involved in the electrochemical reaction, 50 μmol/L capsaicin solution was scanned under different pH (1, 2, 3, 4, 5, 6, 7, and 8) using the CV method [10]. A total of 0.1 mol/L of HCl solution was used for the supporting electrolyte of pH = 1, and PBS solution was used for supporting electrolytes of pH = 2–8. To examine the enhanced electrochemical response of capsaicin, 50 μmol/L capsaicin solution was scanned by CV and DPV method with GCE, GCNT, β-GCE, and β-GCNT [31]. To verify whether other capsaicinoids had the same electrochemical response as capsaicin, 50 μmol/L capsaicin and dihydrocapsaicin solution were scanned by CV method [6]. All experiments were carried out three times.

### 2.4. Optimization of DP-AdSV Parameters

In order to obtain the most optimal parameters of DP-AdSV, 50 μmol/L of capsaicin solution was used as the detection object. The initial experimental parameters of the DP-AdSV method involved a deposition time of 120 s, a deposition potential of −100 mV, a potential range of 200 mV~900 mV, an impulse amplitude of 50 mV, a pulse width of 200 ms, a pulse period of 400 ms, an equilibrium time of 2 s, an initial supporting electrolyte solution of 0.1 mol/L of HCl solution, and the use of β-GCNT as the working electrode. 

To obtain the optimal supporting electrolyte solution, the support electrolytes of different pH were used. A total of 0.1 mol/L of HCl solution was used as the supporting electrolyte of pH = 1, and PBS solution was used as the other supporting electrolytes. To optimize the deposition time, the other parameters were fixed at the initial value, and the deposition time was changed to 5 at 180 s. After this was concluded, to optimize the deposition potential, the deposition time was fixed at the optimal value and the other parameters were fixed at the initial value, then the deposition potential was changed at −400 to 200 mV. To eliminate the matrix effect, samples with different dilution degrees (undiluted, 5 times dilution, and 10 times dilution) were tested using DP-AdSV [6]. All experiments were carried out three times.

### 2.5. Performance Evaluation of the Proposed Sensor

To obtain the limit of detection (LOD), the DP-AdSV curves of different capsaicin concentrations (0.5, 1.0, 5.0, 10.0, 25.0, 40.0, 50.0, 60.0, 75.0, and 100.0 μmol/L) were detected using DP-AdSV under optimum conditions [32,33]. To investigate the reproducibility of β-GCNT, 50 μmol/L capsaicin was detected using five electrodes under the optimum conditions of DP-AdSV [32]. To investigate the stability of β-GCNT, β-GCNT was stored at 4 ℃ for 20 days and 50 μmol/L capsaicin solution was detected every 10 days using β-GCNT under the optimum conditions of DP-AdSV [32]. To study the interference, cholesterol (1 μg/mL), glucose (500 μmol/L), sucrose (500 μmol/L), and inorganic salt ions (0.01 mol/L of Mg^2+^, K^+^, Na^+^, Ca^2+^, Cl^−^ and SO_4_^2−^) were added to 50 μmol/L capsaicin solution and the doped capsaicin solution was detected under the optimum conditions of DP-AdSV [6]. All experiments were carried out three times. 

### 2.6. Application of Actual Sample Detection

The preparation of actual test samples (soy sauce and pot-roast duck neck, soy sauce and pot-roast chicken claw, and soy sauce and pot-roast beef) was as follows: 5 g of sample was placed into a large beaker after crushing. Then, 20 mL of methanol solution (80%) and 0.2 mL of zinc acetate solution (220 g/L) were added to the beaker. After ultrasound at 60 ℃ for 30 min, the beaker was placed in a refrigerator at −20 ℃ and cooled for 15 min. Then, the filtrate solution was collected using filter paper. Next, the extraction was repeated twice and all filtrates were collected together. Finally, the solution was metered to 50 mL for standby using methanol solution (80%). The solution was filtered using 0.22 μm filter membrane before detection. 

To obtain the recovery rates of the actual sample, different concentrations (5, 10, and 20 μmol/L) of capsaicin and dihydrocapsaicin standard solution were added to the samples and three parallel samples were prepared for each concentration. All samples were detected three times using the DP-AdSV method under optimum conditions [6,32].

### 2.7. Statistical Analysis

The raw data was exported from the electrochemical workstation to the Excel component of MS Office. Then, these data were sorted, processed, and imported into the OriginPro8.0 to draw the graphics.

## 3. Results and Discussion

### 3.1. Characterization of Sensors

#### 3.1.1. SEM Characterization

**Figure 1:**
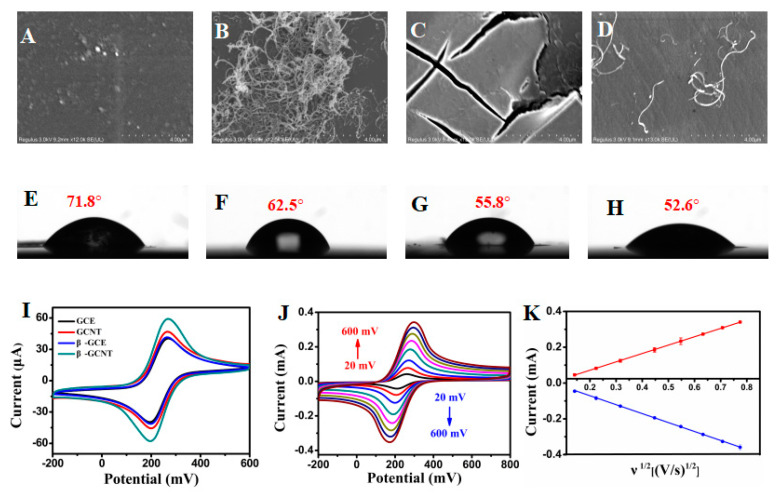
Characterization results of different sensors. SEM images of GCE (**A**), GCNT (**B**), β-GCE (**C**), and β-GCNT (**D**); static contact angles on the GCE (**E**), GCNT (**F**), β-GCE (**G**) and β-GCNT (**H**); CV curves of 10 mmol/L [Fe(CN)_6_]^3−/4−^ on GCE, GCNT, β-GCE and β-GCNT (**I**); CV curves of 10 mmol/L [Fe(CN)_6_]^3−/4−^ on β-GCNT under different scanning rates (**J**); the linear fitting of scanning rate square root to redox peak current value (**K**). All electrochemical experiments were carried out three times and representative curves were selected.

The surface morphology of GCE, GCNT, β-GCE, and β-GCNT sensors were shown in Figure 1. It could be clearly seen that the surface of bare GCE was smooth (Figure 1A), while different substances existed on the GCNT, β-GCE, and β-GCNT surfaces (Figure 1B–D), preliminarily indicating the success of the preparation of different sensors [31,32]. In the case of β-GCE, a thin film existed on its surface (Figure 1C), probably formed by the crystallization of β-CD after electrode drying. For GCNT and β-GCNT, it is worth noting that MWCNT-COOH twined together without any order on the GCNT surface (Figure 1B), while MWCNT-COOH could disperse relatively on β-GCNT’s surface (Figure 1B), indicating that β-CD could improve the dispersion of MWCNT-COOH on GCE surface [31].

#### 3.1.2. Static Contact Angles Characterization

Static contact angles of GCE, GCNT, β-GCE, and β-GCNT sensors are also shown in Figure 1. It was noted that bare GCE had a relatively hydrophilic surface with a contact angle of about 71.8° (Figure 1E). After being modified with MWCNT-COOH and β-CD, the hydrophilicity of the surface was obviously enhanced, and the contact angles were 62.5° and 55.8°, respectively (Figure 1F,G), indicating both β-CD and MWCNT-COOH could improve hydrophilicity of the sensor surface. Compared with GCNT and β-GCE, the surface of β-GCNT was more hydrophilic with a contact angle of about 52.6° (Figure 1H). Previous reports indicated that the relatively hydrophilic surface could facilitate electron exchange in electrochemical process [31]. Therefore, the above results not only further illustrated the success of β-GCNT sensor fabrication, but also provided another explanation for the performance enhancement mechanism of the β-GCNT sensor.

#### 3.1.3. Electrochemical Characterization

The electrochemical characterization of GCE, GCNT, β-GCE, and β-GCNT sensors was studied by CV scanning of [Fe(CN)_6_]^3−/4−^. As shown in Figure 1I, the order of the peak current value was β-GCNT > GCNT > β-GCE > GCE. Meanwhile, the redox peak current of [Fe(CN)_6_]^3−/4−^ on β-GCNT increased significantly as the scanning rate increased and featured a good linear relationship with the square root of the scanning rate (Figure 1J,K). These findings demonstrated that the composites composed of β-CD and MWCNT could efficiently promote electron transfer with a diffusion-controlled process [10]. It was worth noting that the peak current of β-GCNT was obviously enhanced in comparison to GCNT and the peak current of β-GCE showed little increase in comparison to GCE. Combined with the results of SEM and static contact angles, it was preliminarily inferred that the reason for the excellent performance of β-GCNT was more that β-CD made MWCNT-COOH more dispersed on the surface of β-GCNT. Contrary to previous studies [28], the direct improvement of sensor performance by dropping the β-CD solution was negligible, which might be why the preparation method of β-CD electrochemical sensors used in most previous studies was electrodeposition method [28]. 

### 3.2. Electrochemical Behavior of Capsaicinoids

#### 3.2.1. The Electrochemical Reaction Process of Capsaicin

**Figure 2:**
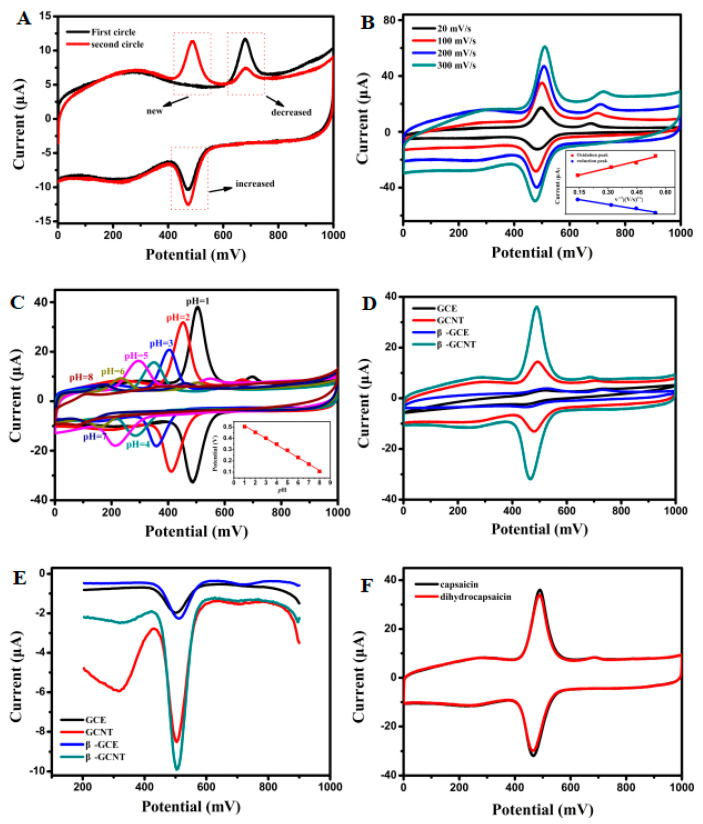
Study on the electrochemical behavior of capsaicinoids. First circle and second circle CV curves of 50 μmol/L capsaicin in 0.1 mol/L HCl solution on β-GCNT (**A**); the effects of scanning rate (**B**) and pH (**C**) on the CV behavior of 50 μmol/L capsaicin in 0.1 mol/L HCl solution on β-GCNT; the CV curves (**D**) and DPV curves (**E**) of 50 μmol/L capsaicin in 0.1 mol/L HCl solution on GCE, GCNT, β-GCE and β-GCNT; The CV cueves of 50 μmol/L capsaicin and dihydrocapsaicin in 0.1 mol/L HCl solution (**F**). All electrochemical experiments were carried out three times and representative curves were selected.

The electrochemical process of capsaicin on β-GCNT sensors was studied by CV scanning of capsaicin solution. The CV curves of first circle and second circle were shown in Figure 2A. An oxidation peak was at approx. 680 mV and a reduction peak was at approx. 470 mV when scanning the first circle. However, when scanning the second circle, the current of the oxidation peak at approx. 680 mV decreased significantly, the peak current of the reduction peak at approx. 470 mV increased obviously, and a new oxidation peak occurred at approx. 486 mV. According to the results, it was preliminarily inferred that capsaicin generated a new species at the first scan, and then the new species continued to undergo electrochemical reaction, which was consistent with previous studies [6]. 

In order to further elucidate the reaction process of capsaicin on β-GCNT sensors, the effects of scanning rate and pH on the CV behavior of capsaicin were investigated. Figure 2B displayed the CV curves of capsaicin under different scanning rates. The redox peak current of capsaicin on β-GCNT increased significantly with the increase in scanning rate and featured a good linear relationship with the square root of the scanning rate. These findings demonstrated that both the oxidation peak at approx. 486 mV and reduction peak at approx. 470 mV were controlled by diffusion [31]. Meanwhile, the electron transfer number of the above reversible peak was calculated to be about two, according to Laviron’s theory [10,33]. Figure 2C illustrated the CV curves of capsaicin under different pH values. The redox peak potential of capsaicin was correlated with pH and it shifted negatively with the increase in the pH value, suggesting that the electrochemical reaction process required proton participation [10]. The number of protons involved in the above reversible peak was calculated to be about 2 according to the linear equation of peak potential and pH [33].

Based on previous reports [6,15,16,17] and the above experimental results, the electrochemical reaction of capsaicin on β-GCNT was initially concluded as follows. First, capsaicin generated a new compound that was more easily oxidized and a reduction product on the β-GCNT sensor surface, which might correspond to the oxidation of the phenolic hydroxyl group or the hydrolysis of the methoxy group [6,16]. Second, the newly formed oxides in the above process continued to undergo reversible redox, which might correspond to the redox reaction of o-phenol structure and o-benzoquinone structure [6,16]. This was basically the same as the mechanism of capsaicin on unmodified screen-printed carbon electrodes in the previous study [6].

#### 3.2.2. The Response Intensity of Different Sensors for Capsaicin

The CV and DPV curves of 50 μmol/L capsaicin in 0.1 mol/L HCl solution on different sensors were shown in Figure 2D,E. It was obvious that the electrochemical response to the β-GCNT was much stronger than that to the GCNT, β-GCE and GCE, indicating that the combination of β-CD and MWCNT-COOH enhanced the sensor’s ability to detect capsaicin. In addition, it was worth noting that the peak current of β-GCNT was obviously enhanced in comparison to GCNT, while there was little difference between β-GCE and GCE. This was consistent with the electrochemical behavior of [Fe(CN)_6_]^3−/4−^, which further confirmed the correctness of the above enhancement mechanism inference.

#### 3.2.3. CV Response of Capsaicin and Dihydrocapsaicin

The electrochemical behavior of capsaicinoids was further investigated by the CV method. The same concentration of capsaicin and dihydrocapsaicin were detected under the same parameters. As shown in Figure 2F, capsaicin and dihydrocapsaicin had similar CV curves. Therefore, it was preliminarily speculated that capsaicinoids had the same electrochemical behavior [6]. Therefore, capsaicin content could be used as a representative for the detection of capsaicinoids content.

### 3.3. Optimization of Experimental Conditions

Taking the peak current value of DP-ADSV as the index, the performance of the sensor for detecting capsaicin was optimized by changing the experimental conditions (supporting electrolyte solution, deposition potential, and deposition time).

#### 3.3.1. Selection of Supporting Electrolyte Solution

**Figure 3:**
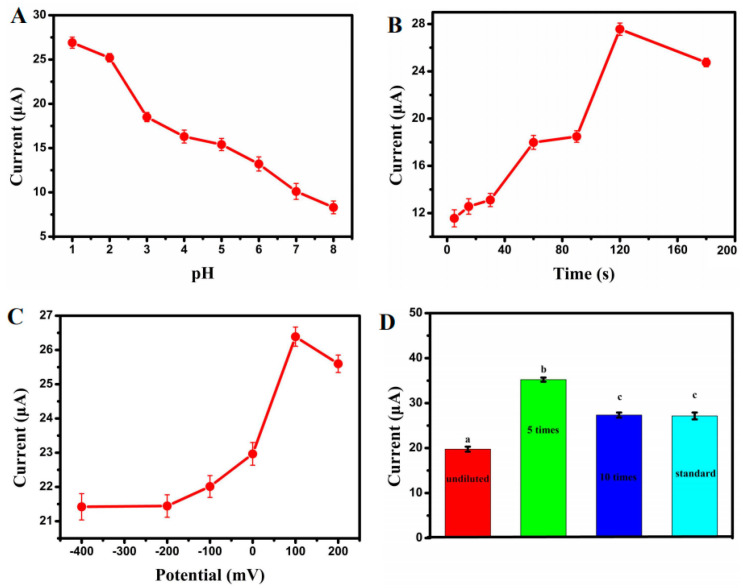
Optimization of supporting electrolyte solution (**A**), deposition time (**B**), deposition potential (**C**), and dilution degree (**D**). 50 μmol/L capsaicin solution was chosen as the study subject and all vertical coordinates about current are absolute values of the actual current.

The electrochemical behavior of capsaicin was significantly affected by the pH value of the supporting electrolyte solution [16]. As shown in Figure 3A, the peak current value of capsaicin was the highest when HCl solution (pH = 1.0) was used as the electrolyte solution. In addition, it could be seen that the response current value of DP-AdSV decreased with the increase in pH, which might be because the electrochemical reaction required the participation of protons [6]. Therefore, HCl solution (pH = 1.0) was selected as the supporting electrolyte solution. 

#### 3.3.2. Parameters Optimization of AdSV

Deposition time and deposition potential were two important parameters of AdSV [6]. As shown in Figure 3B, the peak current of capsaicin increased rapidly when the deposition time was less than 120 s and the peak current decreased when the deposition time was greater than 120 s. The reason might be that when the concentration of capsaicin on the surface of the sensor was low, the higher the concentration of capsaicin, the stronger the electrochemical response, but when there was more capsaicin on the surface of the sensor, excessive capsaicin would block the reaction site on the surface of the sensor, thus affecting the electrochemical reaction [32]. Therefore, 120 s was selected as the optimized deposition time. As shown in Figure 3C, the peak current increased when the range of deposition potential was between −400 and 100 mV and the peak current decreased when the deposition potential was between 100 and 200 mV, which might be caused by the effect of deposition potential on the charge on the electrode surface. Therefore, 100 mV was selected as the deposition potential. Meanwhile, these results also indicated that the combination of DPV and ADSV could significantly enhance the electrochemical response of capsaicin.

#### 3.3.3. Matrix Effects

The effect of matrix on electrochemical detection of capsaicin was investigated by series dilution of the prepared samples using supporting electrolyte solution. The extraction solution of soy sauce and pot-roast duck neck was selected as the research object. As shown in Figure 3D, the peak current of undiluted samples was lower compared with the same concentration of capsaicin standard solution, which might be because the undiluted sample contained many of impurities that could block the reaction site on the surface of the sensor [32]. The peak current of the five times diluted sample rose significantly compared with the same concentration of capsaicin standard solution, which might be because the analog of capsaicin in the sample interfered with the capsaicin detection [6]. Meanwhile, the peak current of 10 times diluted sample was basically consistent with that of the standard solution, indicating the matrix effect could be counteracted by 10 times dilution. Therefore, a 10 times diluted sample was used to eliminate the matrix effect.

### 3.4. Methodological Evaluation

#### 3.4.1. Limit of Detection

**Figure 4:**
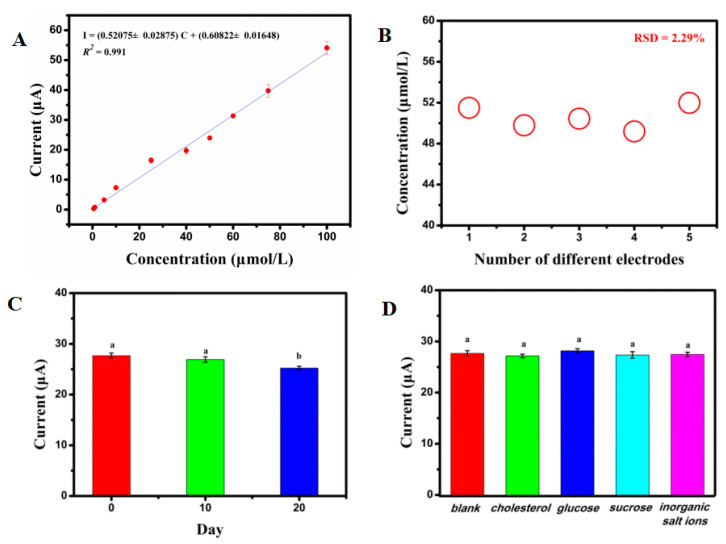
Methodological evaluation of the method proposed in this work. The standard curve of capsaicin in 0.1 mol/L HCl solution on β-GCNT (**A**); the reproducibility (**B**), stability (**C**), and anti-interference (**D**) of the method proposed in this work. All vertical coordinates about current are absolute values of the actual current.

As shown in Figure 4A, the DP-AdSV curves of different capsaicin concentrations detected by β-GCNT under optimum conditions showed that the peak current value of capsaicin increased with the increase in the capsaicin concentration. A standard curve was obtained by linear fitting. The linear equation was *I* (μA) = 0.52075 ± 0.02875 *C* (μmol/L) + 0.60822 ± 0.01648, R^2^ = 0.991 (*I* represented current intensity, and *C* represented the concentration of capsaicin) (*p* < 0.05). The phenomenon provided the possibility for the quantitative detection of capsaicin concentration. The calculation of LOD was based on the method recommended by the International Union of Pure and Applied Chemistry (IUPAC) [6,33]. The results showed that the β-GCNT sensor had a lower LOD of 0.27 μmol/L to capsaicin, which profited from the composites composed of β-CD and MWCNT-COOH being able to efficiently promote the catalytic properties of capsaicin.

#### 3.4.2. Reproducibility and Stability

After capsaicin was detected by β-GCNT, the reactants would be adsorbed on the electrode surface, which would affect the next detection. Therefore, the stability of the sensor could only be achieved by examining the detection performance of different electrodes. As shown in Figure 4B, five different β-GCNT sensors were used to detect 50 μmol/L capsaicin were 51.50, 49.79, 50.44, 49.19, and 51.98 μmol/L, respectively. After calculation, the relative standard deviation (RSD) of five different β-GCNT sensors to capsaicin was 2.29%, indicating the difference among different sensors was within a controllable range [32,33]. As shown in Figure 4C, the peak current value of β-GCNT sensor to capsaicin decreased with the increase in preservation time during storage at 4 °C for 20 days, which could be due to the fact that excessive preservation time led to the slight damage of the composite film composed of β-CD and MWCNT-COOH [32,33]. However, the peak current value of β-GCNT sensor was 91.20% of the initial value after 20 days. Thus, our proposed sensor exhibited satisfactory reproducibility and stability for capsaicin detection.

#### 3.4.3. Anti-Interference Performance

A large amount of cholesterol, sugar and inorganic salt ions exists in soy sauce and pot-roast meat products. Therefore, cholesterol, glucose, sucrose, and common inorganic salt ions were selected as interferers in our work. As shown in Figure 4D, cholesterol (1 μg/mL), glucose (500 μmol/L), sucrose (500 μmol/L) and inorganic salt ions (0.01 mol/L of Mg^2+^, K^+^, Na^+^, Ca^2+^, Cl^−^ and SO_4_^2−^) did not interfere with the peak current value of capsaicin, indicating that these substances generated negligible interference for capsaicin detection [6]. Therefore, the β-GCNT sensor had a good recognition effect on capsaicin.

### 3.5. Validation of Actual Sample Detection

In order to investigate the practicability of the β-GCNT sensor, three kinds of sauce and pot-roast meat products available commercially were tested by the DP-AdSV method. The results are listed in Table 1 and Table 2. The recovery rates of different capsaicin content were between 86.70% and 121.50%, and the recovery rates of different dihydrocapsaicin content were between 83.20% and 136.26%, indicating that the method proposed in our work could accurately detect the content of capsaicinoids in three kinds of products and the error is within the allowable range. Moreover, the relative standard deviation (RSD) of different capsaicin content were lower than 5.50% and the RSD of different dihydrocapsaicin content were lower than 6.85%, indicating that the test data of parallel samples have good stability. To sum up, our proposed electrochemical method could realize trace detection of capsaicinoids content in three kinds of sauce and pot-roast meat products. 

## 4. Conclusions

The determination of capsaicinoids content is the key to the evaluation of the pungent taste degree of sauce and pot-roast meat products. In this work, an electrochemical sensing platform for detecting capsaicinoids content was established with a β-GCNT sensor. The proposed method could determine the total content of capsaicinoids because capsaicin and dihydrocapsaicin had similar electrochemical readings from the β-GCNT sensor. Moreover, the β-GCNT sensor showed an acceptable LOD to detect capsaicinoids content and also successfully realized trace detection of capsaicinoids content in three kinds of sauce and pot-roast meat products. As the electrochemical method has the potential for miniaturization detection, the developed method provides a promising tool in the food industry. This study has important reference value for the standardization of pungent taste degree and flavor control in the food industry.

## Figures and Tables

**Table 1:** Standard addition recovery experiments with different capsaicin content (*n* = 3).

Samples	Added (μmol/L)	Detection (μmol/L)	Recovery Rates (%)	RSD (%)
Soy sauce and pot-roast duck neck	5.00	5.69	113.80	3.15
	10.00	12.15	121.50	4.37
	20.00	18.64	93.20	2.18
Soy sauce and pot-roast chicken claw	5.00	5.88	117.60	5.50
	10.00	10.67	106.70	4.62
	20.00	20.48	102.40	5.15
Soy sauce and pot-roast beef	5.00	5.10	102.00	5.42
	10.00	10.90	109.00	4.25
	20.00	17.34	86.70	3.75

**Table 2:** Standard addition recovery experiments with different dihydrocapsaicin content (*n* = 3).

Samples	Added (μmol/L)	Detection (μmol/L)	Recovery Rates (%)	RSD (%)
Soy sauce and pot-roast duck neck	5.00	4.16	83.20%	4.66
	10.00	11.42	114.09%	3.82
	20.00	23.09	115.48%	4.15
Soy sauce and pot-roast chicken claw	5.00	6.81	136.26%	5.65
	10.00	12.85	128.54%	6.85
	20.00	21.53	107.67%	6.14
Soy sauce and pot-roast beef	5.00	5.28	105.60%	5.72
	10.00	12.48	124.83%	4.38
	20.00	24.11	120.56%	3.95

## Data Availability

Not applicable.
